# Splendor and misery of adaptation, or the importance of neutral null for understanding evolution

**DOI:** 10.1186/s12915-016-0338-2

**Published:** 2016-12-23

**Authors:** Eugene V. Koonin

**Affiliations:** National Center for Biotechnology Information, National Library of Medicine, Bethesda, MD 20894 USA

## Abstract

The study of any biological features, including genomic sequences, typically revolves around the question: what is this for? However, population genetic theory, combined with the data of comparative genomics, clearly indicates that such a “pan-adaptationist” approach is a fallacy. The proper question is: how has this sequence evolved? And the proper null hypothesis posits that it is a result of neutral evolution: that is, it survives by sheer chance provided that it is not deleterious enough to be efficiently purged by purifying selection. To claim adaptation, the neutral null has to be falsified. The adaptationist fallacy can be costly, inducing biologists to relentlessly seek function where there is none.

## The Panglossian paradigm and adaptationist just-so stories

Darwin’s concept of evolution is centered on natural selection, or survival of the fittest [1]. Although Darwin did realize that organisms possess structures and even entire organs that might not have an extant function, as is the case of rudiments [2], on the whole, selectionist thinking has heavily dominated the biological literature ever since. In its extreme but not uncommon form, the selectionist, or adaptationist, paradigm perceives every trait as an adaptation. Under this view of biology, the first and most important question a researcher asks about any structure (including any genomic sequence) is: what is it for? Often, this question is followed up with experiments aimed at elucidating the perceived function.

**Fig. 1:**
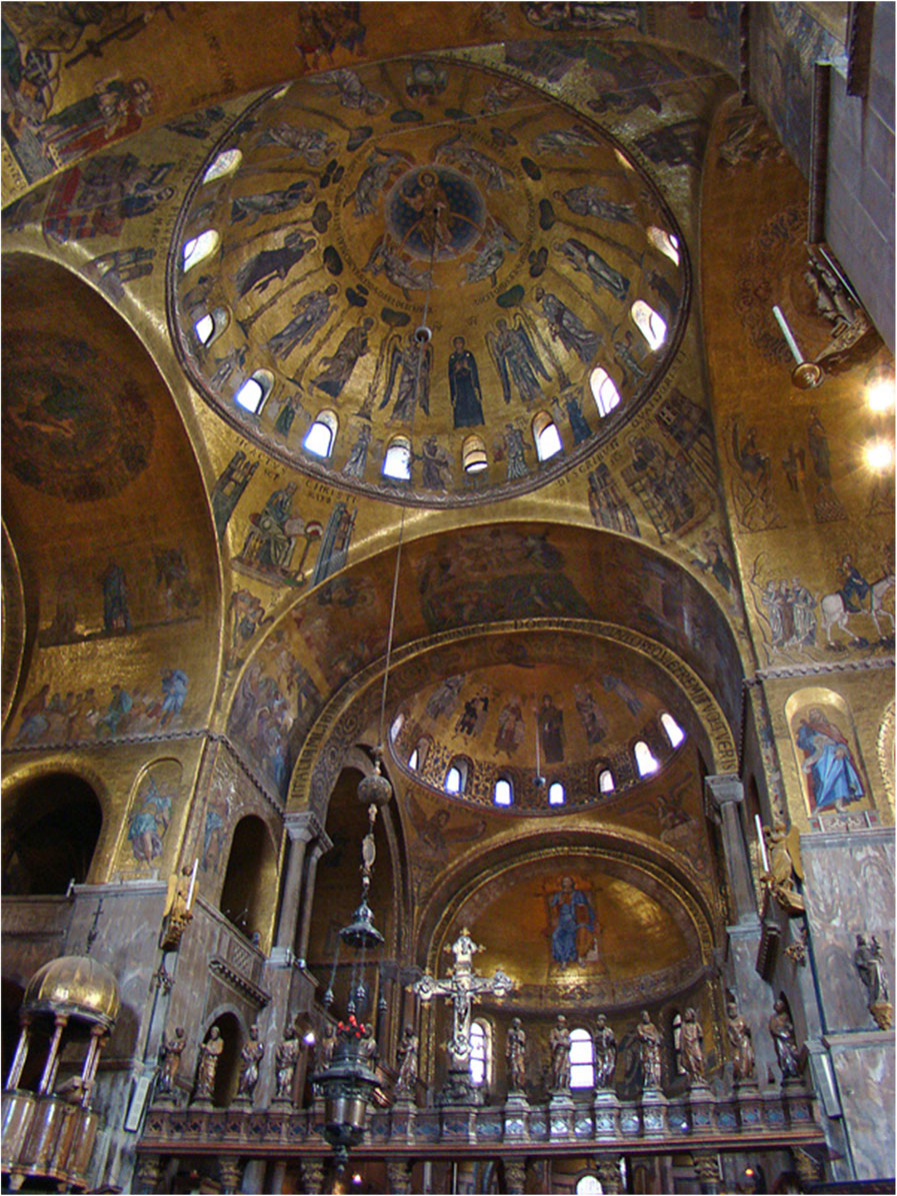
The spandrels of San Marco. The structures that support the arches of the San Marco basilica in Venice are notable for the pictures that decorate them; however, the original role of these structures (spandrels) has nothing to do with the images they carry

**Fig. 2:**
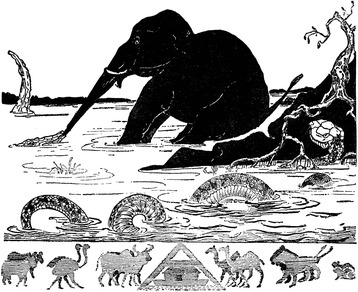
How the elephant got his trunk. An illustration from Rudyard Kipling’s Just So Stories, in which he imagines how striking features of various animals came into being. Here the elephant’s nose is seen being stretched into a trunk as the elephant strains to escape when it is seized by a crocodile. (The actual title of the story is “The elephant’s child”)

Is the pan-adaptationist paradigm valid, especially at the genomic level? In a classic 1979 article [3], unforgettably entitled “The spandrels of San Marco”, Stephen Jay Gould and Richard Lewontin mounted the first all out, frontal attack on pan-adaptationism, which they branded the Panglossian Paradigm after the inimitable Dr. Pangloss of Voltaire’s *Candide ou L’Optimisme* [4], with his “best of all possible worlds”. The argument of Gould and Lewontin is purely qualitative and centers on the metaphorical notion of spandrels, as they denoted biological structures that do not appear to be adaptations per se but rather are necessary structural elements of an organism [5]. The analogy comes from architectural elements that are necessitated by the presence of gaps between arches and rectangular walls, and that can be exploited decoratively to host images, as with the images of archangels and evangelists in the Venetian San Marco basilica (Fig. 1): the spandrels have an essential structural function and by no means have been designed for this decorative purpose. Analogously, biological spandrels can be exapted (recruited) for various functions, although their origin is non-adaptive (exaptation is a new term introduced by Gould and Vrba to denote gain or switch of function during evolution). Rather than hastily concocting adaptationist “just-so stories” (in reference to Rudyard Kipling’s book of lovely tales [6] on how the elephant got his trunk (Fig. 2) and the jaguar his spots—did Kipling actually sense the inadequacy of naïve adaptationism?), submitted Gould and Lewontin, a biologist should attempt to carefully and objectively reconstruct the evolutionary histories of various traits of which many will emerge as spandrels.

Spandrels and exaptation are elegant and biologically relevant concepts but do they actually refute pan-adaptationism? Seemingly not—in particular because clear-cut examples of spandrels are notoriously difficult to come up with. Nevertheless, the essential message of Gould and Lewontin, that telling just-so stories is not the way to explain biology, stands as true and pertinent as ever in the post-genomic era. Let us explore the reasons for this, which could actually be simpler and more fundamental than those envisaged by Gould and Lewontin.

## The fortunes of adaptationism in the (post)genomic era

The adaptationism debate took a new dimension and became far more acute with the realization and subsequent compelling demonstration by genomic sequencing that, at least in the genomes of complex multicellular organisms, the substantial majority of the DNA did not comprise protein-coding sequences. Hence the notion of junk DNA which flew in the face of adaptationist thinking like no other concept before [7–9]. Junk DNA seems to cause a visceral reaction of denial in many if not most biologists, indeed, those that consider themselves “good Darwinists”: how could it be that the majority of the DNA in the most complex, advanced organisms is non-functional garbage? Taken at face value, this possibility seems to defy evolution by natural selection because one would think that selection should eliminate all useless DNA.

The most typical “refutation” of the junk DNA concept involves “cryptic functions” and essentially implies that (almost) every nucleotide in any genome has some functional role—we simply do not (yet) know most of these functions. Recent discoveries of functional genomics and systems biology do add some grist to the adaptationist mill. Although protein-coding sequences comprise only about 1.5% of mammalian genomic DNA, the genome is subject to pervasive transcription—that is, (nearly) every nucleotide is transcribed at some level, in some cells and tissues [10–12]. Moreover, it has been shown that numerous non-coding transcripts are functional RNA molecules, in particular long non-coding RNAs (lncRNAs), that are involved in a variety of regulatory processes [13–15]. All these findings led to “genomic pan-adaptationism”—the view that cryptic functions rule, so that (nearly) all of those transcripts covering the entire genome actually perform specific, elaborate roles that remain to be uncovered by focused experimentation [16–19]. This view has reached its pinnacle in the (in)famous announcement by the ENCODE project of the “functionality of 80% of our genome” [20–23]. In the elegant phrase of Elizabeth Pennisi, the ENCODE project has “written a eulogy for junk DNA” [24].

Genomic pan-adaptationism may be attractive to many biologists, but it faces a formidable problem that was emphasized by several evolutionary biologists immediately after the publication of the striking claims by ENCODE [25–28]. Careful estimates of the fraction of nucleotides in mammalian genomes that are subject to selection, as assessed by evolutionary conservation, produce values of 6 to 9% [29–31]. Allowing some extra for very weakly selected sites, no more than 10% of the genome qualifies as functional, under the key assumption that selection equals functionality [25, 31]. This assumption hardly needs much justification: the alternative is functionality that is not reflected in evolutionary conservation over appreciable time intervals, a contradiction in terms. So the evolutionary estimates of the role of adaptation in shaping complex genomes are a far cry from genomic pan-adaptationism that is deemed compatible with or even a consequence of pervasive transcription. Where do we go from here?

## In the light of population genetics

“Nothing in biology makes sense except in the light of evolution”—arguably, this famous pronouncement of Theodosius Dobzhansky [32, 33] is by now embraced by all biologists (at least at the level of lip service). However, an essential extension to this statement is not nearly as widely recognized. It was formulated by Michael Lynch and goes thus: “Nothing in evolution makes sense except in the light of population genetics” [34]. Yet, without this addition, Dobzhansky’s statement, even if manifestly valid in principle, makes rather little sense in practice. Indeed, population genetic theory serves to determine the conditions under which selection can or cannot be effective. As first shown by Sewall Wright, the evolutionary process is an interplay of selection and random drift, or simply put, fixation of mutations by chance [35, 36]. For adaptive evolution to occur, selection has to be powerful enough to clear the drift barrier [37, 38] (Fig. 2). Without going in detail into the theory, the height of the barrier is determined by the product *N*_*e*_ *s* where *N*_*e*_ is the effective population size and *s* is the selection coefficient associated with the given mutation. If |*N*_*e*_ *s*| > > 1, the mutation will be deterministically eliminated or fixed by selection, depending on the sign of *s.* In contrast, if |*N*_*e*_ *s*| < 1, the mutation is “invisible” to selection and its fate is determined by random drift. In other words, in small populations, selection is weak and only strongly deleterious mutations are weeded out by purifying selection; and conversely, only strongly advantageous mutations are fixed by positive selection. Considering the empirically determined characteristic values of *N*_*e*_ and *s*, these simple relations translate into dramatically different evolutionary regimes depending on the characteristic effective population sizes of different organisms [34, 36, 39].

Simple estimates show that in prokaryotes, with *N*_*e*_ values on the order of 10^9^, the cost of even a few non-functional nucleotides is high enough to make such useless sequences subject to efficient purifying selection that “streamlines” the genome [40]. Hence virtually no junk DNA in prokaryotes, which have “wall-to-wall” genomes composed mostly of protein-coding genes, with short non-coding, intergenic regions. Exceptions are observed only in the genomes of some parasitic bacteria that most likely go through population bottlenecks and thus cannot efficiently purge accumulating pseudogenes due to enhanced drift [41, 42].

**Fig. 3:**
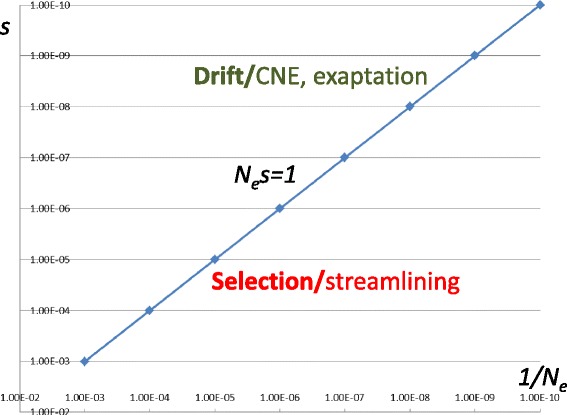
The drift threshold and evolutionary regimes. The *N*_*e*_ *s* = 1 (*s* = 1/*N*_*e*_) line is the drift threshold that separates the domains of the *N*_*e~*_ *s* phase space corresponding to the selection-dominated and drift-dominated evolutionary regimes

The situation is dramatically different in the genomes of multicellular eukaryotes, especially animals, that form small populations, with *N*_*e*_ of about 10^4^ to 10^5^. In these organisms, only strongly deleterious or strongly beneficial mutations, with |*s*| > 10^−4^, clear the drift barrier and accordingly are either eliminated or fixed by selection (Fig. 3). These parameters of the evolutionary regime seem to account for the major genomic features of different organisms, in particular, the baroque genomes of multicellular organisms [36]. Consider one of the most striking aspects of eukaryotic genome organization, the exon–intron gene architecture. Virtually all eukaryotes possess at least some introns, and the positions of many of these have been conserved through hundreds of millions of years [43, 44]. Counterintuitive as this might seem, evolutionary reconstructions in my laboratory clearly indicate that the ancestral state in most major groups of eukaryotes and, apparently, the last common eukaryotic ancestor had an intron density close to that in extant animals [45]. Why have eukaryotes not lost their introns? The adaptationist perspective has a ready “just-so story”: introns perform important biological functions. And indeed, this is the case for quite a few introns that harbor genes for small non-coding RNAs and, less frequently, proteins and are involved in various regulatory roles [46]. Nevertheless, the inconvenient (for adaptationism) fact is that a substantial majority of introns harbor no detectable genes, show no appreciable sequence conservation even in closely related organisms, and, overall, look much like junk [44]. The population-genetic perspective provides concrete indications that this is what they are. Simple estimates taking into account the characteristic values of *N*_*e*_, mutation rate, and the target size for deleterious mutations in splicing signals (only about 25 base pairs per intron) show that purifying selection in typical populations of multicellular eukaryotes is too weak to weed out individual introns [47, 48]. Therefore, the introns persist in eukaryotic genomes simply because, at an early stage of eukaryotic evolution, they invaded the genomes as mobile elements, and subsequently, in many (but by no means all) lineages of eukaryotes, selection was not strong enough to get rid of them. To cope with this inescapable burden, eukaryotes have evolved a global solution, the highly efficient splicing machinery (see next section).

Introns are by no means the only genomic feature that is apparently there just because it can be. Along the same lines, it is easy to show that even duplications of individual genes have limited deleterious effect and fall below the drift threshold in organisms with small *Ne*. The notorious pervasive transcription seems to belong in the same category. The minimal sequence requirements (that is, the selection target) for spurious transcription are less thoroughly characterized than those for splicing but are most likely to be of the same order if not lower, in which case, transcriptional noise simply cannot be eliminated by selection, resulting in pervasive transcription.

## Global vs local selection: adapting to the ineffectiveness of adaptation

A major corollary of the population-genetic perspective on evolution is a dramatic change in the very nature of prevailing evolutionary solutions depending on the power of selection, which is primarily determined by the effective population size. The local solutions that are readily accessible in the strong selection regime, in particular in large populations of prokaryotes—because even features associated with very small *s* values are subject to selection—are impossible in the weak selection regime, that is, in small, drift-dominated populations. This ineffectiveness of local solutions dictates a completely different evolutionary strategy: that is, global solutions that do not eliminate deleterious mutations as they arise, but instead minimize the damage from genomic features and mutations whose deleterious effects are not sufficient to clear the draft barrier in small populations [49, 50]. Introns once again present a perfect example. Because introns cannot be efficiently eliminated by selection, eukaryotes have evolved, first, the highly efficient and precise splicing machinery, and second, multiple lines of damage control such as nonsense-mediated decay, which destroys aberrant transcripts containing premature stop codons [36, 51]. In a more speculative vein, the nucleus itself may have evolved as a damage-control device that prevents the exit of unprocessed transcript to the cytoplasm [52, 53]. The elaborate global solutions for damage control are by no means limited to introns. For example, the germline expression of transposons, a class of genomic parasites that under weak selection cannot be efficiently eliminated, is suppressed by the piRNA systems, a distinct branch of eukaryotic RNA interference [54]. The switch from local to global solutions necessitated by the ineffectiveness of selection in small populations signifies a major shift in the character of adaptation: under this evolutionary regime, much of adaptation involves overcoming such ineffectiveness.

## Subfunctionalization, constructive neutral evolution, and pervasive exaptation

Paradoxical as this may seem, the weak evolutionary regime promotes evolution of phenotypic complexity. Precisely because many genomic changes cannot be efficiently eliminated, routes of evolution that are blocked under strong selection open up. Consider evolution by gene duplication, the mainstream route of evolution in complex eukaryotes [55]. In prokaryotes, duplications are rarely fixed because the deleterious effect of a useless gene-size sequence is sufficient to make them a ready target for purifying selection, since being identical, gene duplicates are useless immediately after duplication except in rare cases of beneficial gene dosage effects. By contrast, in eukaryotes, duplicates of individual genes cannot be efficiently eliminated by selection and thus often persist and diverge [56–59]. The typical result is subfunctionalization, whereby the gene duplicates undergo differential mutational deterioration, losing subsets of ancestral functions [60–62]. As a result, the evolving organisms become locked into maintaining the pair of paralogs. Subfunctionalization underlies a more general phenomenon, denoted constructive neutral evolution (CNE) [63–66]. CNE involves fixation of inter-dependence between different components of a complex system through partial mutational impairment of each of them. Subfunctionalization of paralogs is a specific manifestation of this evolutionary modality. The CNE seems to underlie the emergence of much of the eukaryotic cellular complexity, including hetero-oligomeric macromolecular complexes such as the proteasome, the exosome, the spliceosome, the transcription apparatus, and more. The prokaryotic ancestors of each of these complexes consist of identical subunits that are transformed into hetero-oligomers in eukaryotes as illustrated by comparative genomic analysis from my laboratory, among others [67], conceivably because of relaxation of selection that enables CNE.

Another major phenomenon that shapes the evolution of complexity is pervasive recruitment of “junk” genetic material for diverse functions. There are, of course, different kinds of junk in genomes [28]. Exaptation of parts of mobile genetic elements (MGE) is one common theme. Sequences originating from MGE are routinely recruited for regulatory functions in eukaryotic promoters and enhancers [68–70]. In addition, MGE genes have been recruited for essential functions at key stages of eukaryotic evolution. Striking examples include telomerase and the essential spliceosomal subunit Prp8, both of which originate from the reverse transcriptase of group II self-splicing introns [71], the major animal developmental regulator Hedgehog that derives from an intein [72], and the central enzyme of vertebrate adaptive immunity, the RAG1-RAG2 recombinase that evolved from the transposase of a Transib family transposon [73, 74].

**Fig. 4:**
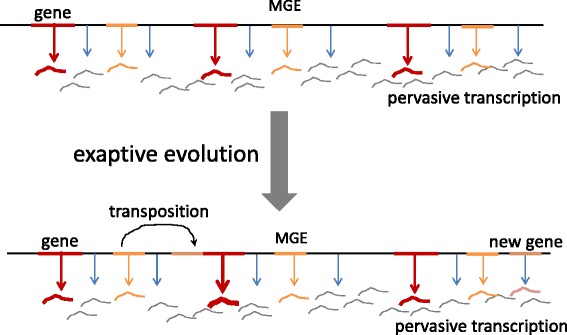
The routes of exaptation. The cartoon schematically shows two types of evolutionary events: exaptation of a function-less transcript that becomes, for example, a lncRNA and exaptation of a MGE that becomes, after transposition, a regulatory region of a pre-existing gene. The thickness of the *arrows* denotes the increase in expression level that is assumed to occur after exaptation

Apart from MGE, the numerous “junk” RNA molecules produced by pervasive transcription represent a rich source for exaptation from which diverse small and large non-coding RNAs and genes encoding small proteins are recruited (Fig. 4) [75, 76]. Actually, the two sources for the recruitment of new functional molecules strongly overlap given the conservative estimates of at least half of the mammalian genome and up to 90% of plant genomes deriving from MGE [77].

These routes of exaptation that appear to be central to eukaryotic evolution notably deviate from Gould’s and Lewontin’s original spandrel concept [3, 5] (Fig. 4). The spandrels of San Marco and their biological counterparts are necessary structural elements that are additionally used (exapted) for other roles, such as depicting archangels and evangelists. The material that is actually massively recruited for diverse functions is different in that it is not essential for genome construction but rather is there simply because it can be, that is, because selection is too weak to get rid of it. Using another famous metaphor, this one from Francois Jacob [78, 79], evolution tinkers with all this junk, and a small fraction of it is recruited, becoming functional and hence subject to selection [76]. The term exaptation may not be the best description of this evolutionary process but could perhaps be retained with an expanded meaning.

The extensive recruitment of “junk” sequences for various roles calls for a modification to the very concept of biological function [76]. Are the “junk” RNA sequences resulting from pervasive transcription non-functional? In the strict sense, yes, but they are endowed with potential, “fuzzy” functional meaning and represent the reservoir for exaptation (Fig. 4). The recruitment of genes from MGE represents another conundrum: these genes encoding active enzymes certainly are functional as far as the MGE is concerned but not in the context of the host organism; upon recruitment, the functional agency switches.

The pervasive exaptation in complex organisms evolving in the weak selection regime appears as a striking paradox: the overall non-adaptive character of evolution in these organisms enables numerous adaptations which ultimately lead to the dramatic rise in organismal complexity [39]. In a higher abstraction plane, though, this is a phenomenon familiar to physicists: entropy increase begets complexity by creating multiple opportunities for the evolution of the system [80, 81].

## Changing the null model of evolution

The population genetic perspective calls for a change of the null model of evolution, from an unqualified adaptive one to one informed by population genetic theory, as I have argued elsewhere [82, 83]. When we observe any evolutionary process, we should make assumptions on its character based on the evolutionary regime of the organisms in question [34]. A simplified and arguably the most realistic approach is to assume a neutral null model and then seek evidence of selection that could falsify it. Null models are standard in physics but apparently not in biology. However, if biology is to evolve into a “hard” science, with a solid theoretical core, it must be based on null models, no other path is known. It is important to realize that this changed paradigm by no means denies the importance of adaptation, only requires that it is not taken for granted. As discussed above, adaptation is common even in the weak selection regime where non-adaptive processes dominate. But the adaptive processes change their character as manifested in the switch from local to global evolutionary solutions, CNE, and pervasive (broadly understood) exaptation.

The time for naïve adaptationist “just so stories” has passed. Not only are such stories conceptually flawed but they can be damaging by directing intensive research toward intensive search for molecular functions where there is none. However, science cannot progress without narratives, and we will continue telling stories, whether we like it or not [83]. The goal is to carefully constrain these stories with sound theory and, certainly, to revise them as new evidence emerges. To illustrate falsification of predictions coming out of the population genetic perspective, it is interesting to consider the evolution of prokaryotic genomes. A straightforward interpretation of the theory implies that under strong selection, genomes will evolve by streamlining, shedding every bit of dispensable genetic material [47]. However, observations on the connection between the strength of purifying selection on protein-coding genes and genome size flatly contradict this prediction: the strength of selection (measured as the ratio of non-synonymous to synonymous substitution rates, *dN/dS*) and the total number of genes in a genome are significantly, positively correlated, as opposed to the negative correlation implied by streamlining [84]. The results of mathematical modeling of genome evolution compared with genome size distributions indicate that, in the evolution of prokaryotes, selection actually drives genome growth because genes acquired via horizontal transfer are, on average, beneficial to the recipients [85]. This growth of genomes is limited by diminishing returns along with the deletion bias that seems to be intrinsic to genome evolution in all walks of life [86]. Thus, a major prediction of the population genetic approach is refuted by a new theoretical development pitted against observations. This result does not imply that the core theory is wrong, rather that specific assumptions on genome evolution, in particular those on characteristic selection coefficient values of captured genes, are unwarranted. Streamlining is still likely to efficiently purge true function-less sequences from prokaryotic genomes.

The above example may carry a general message: the population genetic theory replaces adaptationist just-so stories with testable predictions, and research aimed at falsification of these can improve our understanding of evolution. We cannot get away from stories but making them much less arbitrary is realistic. Furthermore, although most biologists do not pay much attention to population genetic theory, the time seems to have come for this to change because, with advances in functional genomics, such theory becomes directly relevant for many directions of experimental research.

## Abbreviations

CNE: Constructive neutral evolution
MGE: Mobile genetic element
